# Diet & Nutrition: Hyperactive Ingredients?

**DOI:** 10.1289/ehp.115-a578

**Published:** 2007-12

**Authors:** Julia R. Barrett

The question of whether food additives such as preservatives, artificial flavorings, and artificial colorings trigger hyperactivity has been debated for more than 30 years. Research generally has not supported food additives as influencing hyperactivity—whose characteristics include overactivity, inattention, and impulsive behaviors, traits that in extreme forms define attention deficit/hyperactivity disorder (ADHD)—but some studies have found small effects. Most recently, a study published 3 November 2007 in *The Lancet* suggests that the preservative sodium benzoate and commonly used artificial food colorings in fact may exacerbate hyperactive behavior in young children.

In the *Lancet* study, researchers led by Jim Stevenson, a professor of psychology at the University of Southampton, United Kingdom, built upon a previous double-blind placebo-controlled study of preschool children. In that study, published in the June 2004 *Archives of Disease in Childhood*, 3-year-old children on a diet free of artificial dyes and benzoate preservatives exhibited increased hyperactivity when challenged with a drink containing a mixture of the widely used sodium benzoate plus the dyes Sunset Yellow, carmoisine, tartrazine, and Ponceau 4R (in their later paper, Stevenson and colleagues termed this combination “mix A”). Again using a double-blind placebo-controlled design, the Southampton team expanded the study group to include 153 3-year-olds and 144 8- and 9-year-olds representative of the general population.

Children ate diets free of the elements in mix A and a second, more concentrated mixture of additives (“mix B,” comprising sodium benzoate plus the dyes Sunset Yellow, carmoisine, Quinolone Yellow, and Allura Red AC) for six weeks. During that time, they drank a daily serving of plain juice (placebo) or juice containing one of the two mixes; the test drink changed weekly. To measure hyperactivity, the team calculated a global hyperactivity aggregate (GHA) based upon questionnaires completed by parents, teachers, and trained observers. Older children also completed a computer-based assessment of attention. Small but significant increases in GHA occurred with mix A in both age groups, with 3-year-olds showing a greater effect. Mix B was associated with a small significant effect in 8- and 9-year-olds, but not in 3-year-olds, who had a wide range of individual responses.

“The outstanding feature of the results was the similar pattern of an adverse effect across both ages for both mixes—although this did not reach statistical significance in every case,” says Stevenson. An unpublished study based on genetic samples from the children examines these individual differences in greater detail. “Our [forthcoming] data indicate that genetics rather than anything else accounts for these individual differences in response within an age group,” Stevenson says.

Although the Southampton researchers conclude that their results strongly support a relationship between food additives and behavior, they do not claim that food additives cause clinically defined ADHD. “It is very important to clarify that the food additives and preservative studied only increased activity level modestly,” says Andrew Adesman, chief of developmental and behavioral pediatrics at Schneider Children’s Hospital in New Hyde Park, New York. “I think the reasonable lessons from this study are that there may be modest effects on activity level from additives or preservatives and that better, more precise studies are needed to determine whether it is the additives alone, the preservatives alone, or the combination that is responsible for these modest adverse effects.” The authors describe these research needs with the additional requirement of considering the time elapsed between additive consumption and subsequent behavior.

Nevertheless, after reviewing the Southampton study, the British Committee on Toxicity concluded that the results could be clinically relevant for individual children, particularly those who already show a tendency toward hyperactivity. On the basis of this study the British Food Standards Agency, which funded the research, has advised parents to consider eliminating the colorings used in the study from the diets of children who exhibit hyperactive behaviors.

“It will be interesting to see how the [U.S.] Food and Drug Administration reacts to this study,” says Adesman. “Hopefully, they will either encourage or mandate additional studies looking at food effects of additives on children and also adults.”

The FDA is aware of the Southampton study but has not received the study data, according to administration spokesman Mike Herndon. “We will examine this recent report to see if the results suggest whether any action to modify our current regulations is appropriate,” he says. “However, we have no reason at this time to change our conclusions that the ingredients that were tested in this study that currently are permitted for food use in the United States are safe for the general population.”

## Figures and Tables

**Figure:**
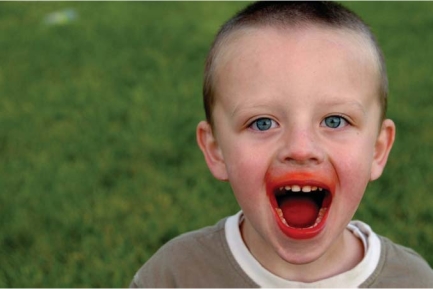
Dyeing for more data The question of whether food additives such as colorings contribute significantly to hyperactivity remains open.

